# EVALUATION OF THE RADIOISOTOPIC SYNOVECTOMY PROTOCOL IN PATIENTS WITH HEMOPHILIA

**DOI:** 10.1590/1413-785220263402e291382

**Published:** 2026-05-11

**Authors:** Marcela dos Santos Martins, Janaina Bosso da Silva Ricciardi, Allan de Oliveira Santos, Margareth Castro Ozelo, Rodrigo Gonçalves Pagnano, Emerson Taro Inoue Sakuma

**Affiliations:** 1 Universidade Estadual de Campinas (UNICAMP), Faculdade de Ciencias Medicas, Sao Paulo, SP, Brazil.

**Keywords:** Hemophilia, Synovectomy, Radioisotopes, Joint diseases, Hemofilia, Sinovectomia, Radioisótopos, Artropatia

## Abstract

**Introduction:**

Hemophilia is an inherited hemorrhagic disorder in which the most common musculoskeletal manifestation is intra-articular hemorrhage. Successive hemarthroses result in chronic synovitis, joint surface destruction, and chronic hemophilic arthropathy. When conservative treatment cannot halt an already established synovitis, removing the synovium may interrupt the process of joint destruction, and radioisotopic synovectomy (RS) becomes one of the treatments of choice.

**Objective:**

To evaluate the safety and effectiveness of the RS protocol in reducing the incidence of hemarthrosis in patients with hemophilia.

**Materials and Methods:**

This retrospective study is based on a review of medical records. We compared the number of bleeding events one year before and one year after RS. Results were submitted to statistical analysis and compared using the Wilcoxon signed-rank test.

**Results:**

65 RS procedures were performed between 2008 and 2018 in patients followed at our institution. The number of bleeding events recorded in the joints submitted to intervention in the year preceding the procedure was 367. In contrast, the number in the following year was 203, a reduction of 44.68% (P<0.001). There were no complications related to the procedure during the analyzed period.

**Conclusion:**

The RS protocol was safe and effective in reducing the number of bleeding events. **Level of Evidence IV; Case Series**.

## RESUMO

**Introdução:**

A hemofilia é um distúrbio hemorrágico hereditário cuja manifestação musculoesquelética mais comum é a hemorragia intra-articular. Sucessivas hemartroses resultam em sinovite crônica, destruição da superfície articular e artropatia hemofílica crônica. Nos casos em que o tratamento conservador não controla a sinovite já instalada, a remoção da sinóvia interrompe o processo de destruição articular, sendo a sinovectomia radioisotópica (SR) um dos tratamentos de escolha.

**Objetivo:**

Avaliar a eficácia e segurança do protocolo de SR adotado em nossa instituição na redução da incidência de hemartroses em pacientes hemofílicos.

**Métodos:**

Estudo retrospectivo baseado em revisão de prontuários. Foi comparado o número de episódios de sangramento um ano antes e um ano após a SR. Os resultados foram submetidos a análise estatística e comparados de acordo com o Wilcoxon signed rank test.

**Resultados:**

Foram avaliadas 65 SR entre os anos de 2008 a 2018. O número de sangramentos nas articulações submetidas à intervenção no ano que a antecedeu foi de 367. O número de sangramentos no ano seguinte ao procedimento foi de 203, uma redução de 44,68% (P<0,001). Não houve complicações relacionadas ao procedimento no período analisado.

**Conclusão:**

O protocolo de sinovectomia radioisotópica adotado foi seguro e eficaz na redução do número de eventos hemorrágicos intra-articulares. **Nível de Evidência IV; Série de Casos**.

## INTRODUCTION

Hemophilia is an inherited bleeding disorder linked to the X chromosome, classified into hemophilia A (factor VIII deficiency) and hemophilia B (factor IX deficiency). Hemophilia A affects between 1 in 5,000 to 10,000 men, while hemophilia B affects 1 in 25,000 to 30,000 men.^1^ The main symptom is bleeding, with up to 90% of episodes occurring in joints and muscles, often spontaneously.^2^ Repeated episodes of hemarthrosis result in chronic synovitis, joint destruction, and the development of chronic hemophilic arthropathy (CHA), with pain, loss of mobility, and deformity.^3,4^

Prophylactic treatment with factor replacement is the standard to prevent bleeding and avoid joint damage. However, in cases of chronic synovitis refractory to conservative treatment, synovectomy is indicated to interrupt the progression of CHA.^5^ Among the modalities of synovectomy, radioisotopic (RS) stands out for being a minimally invasive, effective, and safe procedure, indicated for target joints that present chronic synovitis.^6^

In RS, radioisotopes such as yttrium-90 and samarium-153, both with favorable properties for synovial ablation, are widely used.^7^ Yttrium-90 and samarium-153 are coupled to larger particles, such as hydroxyapatite, to minimize the risk of the radiopharmaceutical leaking out of the joints, ensuring that its action is restricted to the application site and reducing possible adverse effects.^8^

Studies show that RS significantly reduces the joint inflammatory process and the frequency of hemarthroses, relieving pain and improving the quality of life of patients.^9,10^ This study aimed to evaluate the efficacy and safety of the RS protocol adopted in our institution in reducing the incidence of hemarthroses in hemophilic patients.

## MATERIALS AND METHODS

This retrospective cohort study was approved by the Research Ethics Committee in Human Beings of our institution (CAAE: 15464619.6.0000.5404). Medical records and diaries of patients with hemophilia who underwent radioisotopic synovectomy (RS) between 2008 and 2018 were evaluated using our protocol. Demographic data, type and severity of hemophilia, number of hemarthroses recorded in the year before and after RS, as well as associated comorbidities, were collected.

The RS protocol included the use of yttrium-90 labeled hydroxyapatite (Y90-HYP) and samarium-153 (153Sm-HYP), both provided by the Institute of Energy and Nuclear Research-IPEN (São Paulo, SP), divided into three phases: pre, intra, and post-procedure. In the pre-procedure phase, clinical, radiographic, and scintigraphic evaluations were performed. The procedure was performed with aseptic technique, local anesthesia, and administration of coagulation factor. In the post-procedure phase, care such as analgesia, anti-inflammatories, joint immobilization, and outpatient follow-up were adopted.

Patients with hemophilia A or B who underwent RS at our institution were included, while patients with loss of follow-up, those followed at other centers, and cases of advanced arthropathy were excluded. The number of bleedings was recorded in the patients’ charts and diaries. The statistical analysis was performed using the Wilcoxon test for dependent samples, with significance of p<0.05. The Real Statistics program for Microsoft Excel 2010 was used for the analysis.

### SR Protocol:

Pre-procedure phase: Clinical, radiographic, and scintigraphic evaluation of the affected joint. Patients received a dose of coagulation factor (FVIII or FIX) to prevent hemarthrosis during the procedure.

Procedure phase: The radiopharmaceutical, hydroxyapatite labeled with yttrium-90 (Y90-HYP) or samarium-153 (153Sm-HYP), was injected into the joint under aseptic technique and local anesthesia, followed by the injection of corticosteroid to irrigate the needle path during its withdrawal, thus preventing the leakage of radioactive material to the skin. Before the patient was discharged, monitoring of radioactivity presence on the skin at the puncture site was performed using a swab with gauze, monitored by portable Geiger Müller radiation meters and/or in a curiometer. The procedure was guided by ultrasound in the smaller joints (elbow and ankle) and, when necessary, there was aspiration of synovial fluid to confirm the correct location. The joint was immobilized for 48 hours with compressive bandaging.

Post-procedure phase: Patients were clinically followed on days 2, 4, 7, 14, and 30, with monthly follow-up during the first 12 months. Analgesia (Ibuprofen) and coagulation factor were administered according to protocol. In addition, patients underwent scintigraphy to evaluate the distribution of the radiopharmaceutical and any joint leakage within 72 hours after the intervention. All procedures were performed by the same professionals.

## RESULTS

102 SR were recorded between the years 2008 and 2018 in our service. Sixty-five procedures (n=65) were performed on 42 patients followed at the institution, with regular records in the chart. The other procedures, performed on patients followed by other services, were excluded due to lack of sufficient data.

All 65 treated joints achieved technical procedural success, with no adverse responses described in the medical records. The average age of the patients was 20 years. Most procedures were performed on male patients (n=41), and severe Hemophilia A was the most prevalent subtype of the disease (n=38), followed by severe Hemophilia B (n=3) and moderate Hemophilia A (n=1). Of the procedures, 52.30% were performed on patients who were already on prophylaxis with regular doses of coagulation factor (n=34). Among the comorbidities associated with multiple blood transfusions, six cases of hepatitis C were identified (9.09%).

The joints subjected to SR included: knees (n=32), elbows (n=21), and ankles (n=12) (Table 1), being: left knee (n=20), right knee (n=12), left ankle (n=5), right ankle (n=7), left elbow (n=15), and right elbow (n=6).

**Table 1:** Demographic data of the patients.

Characteristic	Value
Sex	
Male	41
Female	1
Type of hemophilia	
Hemophilia A	39
Hemophilia B	3
Age (average) Target joint	20 years
Knee	32
Elbow	21
Ankle	12

**Figure 1:**
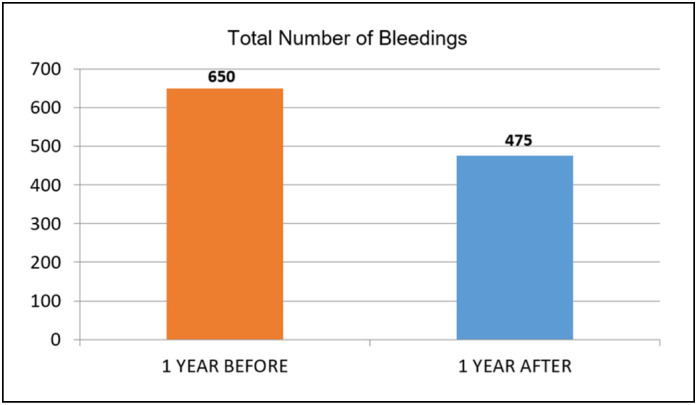
Comparison of the total number of bleedings one year before and one year after the RS.

The total number of bleedings recorded in the year prior to radioisotopic synovectomy was 650, considering all joints. In the year after the RS, 475 bleedings were recorded, a reduction of 26.92% (P = 0.00927) (Figure 1).

**Figure 2:**
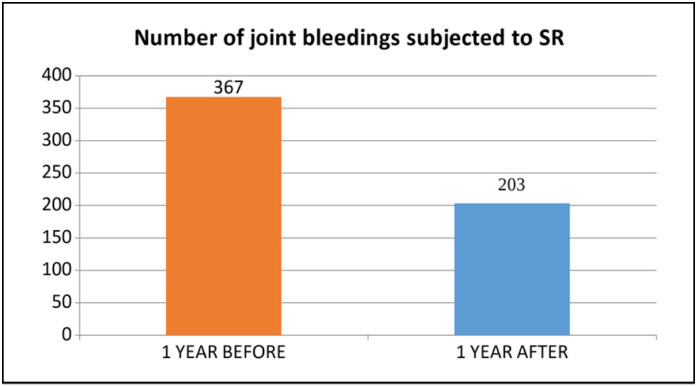
Comparison of the number of bleedings before and after the RS in the treated joints.

Considering only the treated joints, the number of bleedings reduced from 367 in the previous year to 203 in the following year, a reduction of 44.68% (P = 0.000367) (Figure 2).

The average total number of bleedings per patient pre-procedure was 10.0 ± 8.066, while post-procedure it was 7.3 ± 6.995, a reduction of 27.0%. In the treated joints, the average number of bleedings per patient fell from 5.6 ± 5.188 to 3.1 ± 4.072, a reduction of 44.6%.

**Figure 3:**
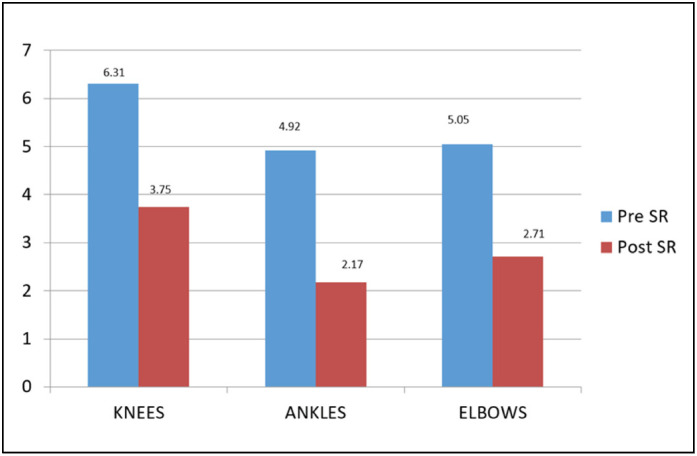
Average bleedings per joint.

In patients treated in the knee (n=32), the number of bleeding episodes pre-RS was 202 (average of 6.31) and post-RS was 120 (average of 3.75), a reduction of 40.57% (P=0.0110). In patients treated in the elbow (n=21), there was a reduction of 46%, from 106 bleeding episodes to 57 (P=0.0328). For the ankle (n=12), the reduction was 55.93%, from 59 to 26 episodes (P=0.0391) (Figure 3 and Table 2).

**Table 2:** Bleeding events per joint, in the period of one year before and one year after the RS.

Joint	Bleedings pre-rs	Bleedings post-rs	Decrease	P
Knee (32)	202	120	40.57%	0.011
Ankle (12)	59	26	55.93%	0.039
Elbow (21)	106	57	46.34%	0.032

**Figure 4:**
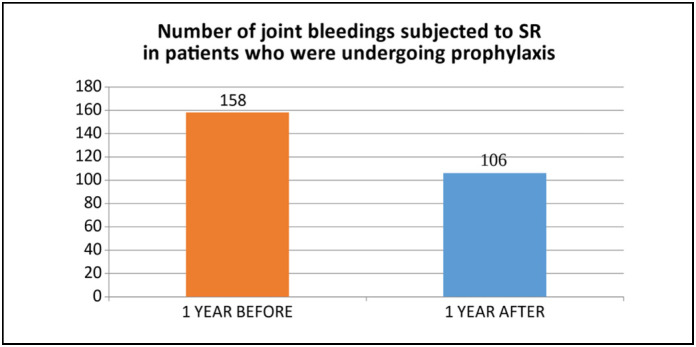
Comparison of bleeding in patients with prophylaxis.

**Figure 5:**
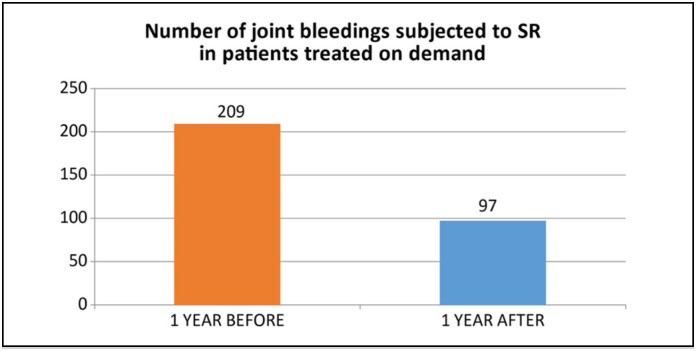
Comparison of bleeding in patients treated with factor on demand.

Patients treated with prophylaxis (n=23) showed a reduction of 32.91% in bleeding in the treated joint (P=0.02) (Figure 4). For patients treated with factor on demand (n=19), the reduction was 53.58% (P=0.001) (Figure 5).

The joints treated with Yttrium-90 were compared to those treated with Samarium-153. Of the 65 joints studied, 32 were treated with Samarium-153 (7 knees, 7 ankles, and 18 elbows) and 30 were treated with Yttrium-90 (23 knees, 4 ankles, and 3 elbows). No records were found regarding the radiopharmaceutical used in 3 joints (2 knees and 1 ankle) of the studied patients.

Among the joints treated with Yttrium-90 (n=30), the number of bleedings was reduced from 200 to 117 (41.5%, P=0.030). In the joints treated with Samarium-153 (n=32), there was a reduction of 45.1%, from 155 to 85 (P=0.006)

## DISCUSSION

Radioisotopic synovectomy (RS) is widely recognized for its safety and efficacy. In addition to the management of hemophilia, its use also extends to the control of synovitis caused by other conditions, such as pigmented nodular synovitis, rheumatoid arthritis, psoriasis, lupus, gout, and ankylosing spondylitis.^11^ After injection, the radiopharmaceutical particles are quickly phagocytized by synovial macrophages and deposited in the outermost layers of the synovial membrane. These particles, due to their size, cannot penetrate the capillary fenestrations, remaining in the joint.^12^ The emission of beta radiation continues for weeks, inducing synovial apoptosis through the production of reactive oxygen species.^11^

The emitted radiation has low penetration power, being mostly absorbed by the synovium, synovial fluid, and superficial layers of cartilage and joint capsule. Adjacent tissues, such as subchondral bone, receive insignificant doses of radiation, which theoretically ensures the safety of the procedure.^12^

Complications related to SR, such as skin necrosis due to extravasation of radiopharmaceuticals, arthritis, and infections, are rarely reported. In the literature, there are reports of two cases of lymphocytic leukemia in children who underwent SR with higher energy tracers, such as gold-198 and phosphorus-32, isotopes that are no longer in current use.^12^ However, the causal relationship between these tracers and the neoplasia has not been well established. Studies with more modern radioisotopes, such as yttrium-90, do not show an increase in the incidence of neoplasms in treated patients.^13^

In our study, no evidence of serious adverse effects, such as skin necrosis or chemical synovitis post-procedure, was found in the reviewed records. These findings suggest that the SR protocol adopted by the Hemocenter of Unicamp is safe, with no early treatment failures identified.

The results of this study also demonstrated the effectiveness of SR in reducing the incidence of hemarthroses in knee, elbow, and ankle joints that underwent SR. It was also possible to compare the effectiveness of SR between patients who received regular prophylaxis and those who received factor on demand. In both groups, SR proved effective in reducing bleeding episodes in the treated joint.

When comparing treatment with different isotopes, yttrium-90 and samarium-153, we observed that both resulted in a reduction in the number of bleedings, demonstrating the effectiveness of both radioisotopes in the treatment of chronic synovitis.

This study has some limitations inherent to retrospective cohorts, such as the absence of a control group, failures in medical record keeping, and the subjectivity of records maintained by the patients themselves. In addition, there is a lack of information on the long-term effects of SR.

It is possible that there was an observation bias, as patients undergoing SR may have paid more attention to the number of bleedings in the treated joint. However, the results indicate that SR promoted a significant reduction in the incidence of hemarthroses in hemophilic patients and proved to be a safe method, with no serious adverse effects reported.

## CONCLUSION

The adopted radioisotopic synovectomy protocol proved to be safe and effective in reducing the number of intra-articular hemorrhagic events over the studied period. No procedure-related adverse events were observed during the follow-up. There was a significant reduction in the number of bleedings in all groups of patients analyzed after the procedure.

## Data Availability

The contents underlying the research are available in the manuscript.
